# Improving Disaster Data Systems to Inform Disaster Risk Reduction and Resilience Building in Australia: A Comparison of Databases

**DOI:** 10.1017/S1049023X2100073X

**Published:** 2021-10

**Authors:** Joseph Cuthbertson, Frank Archer, Andy Robertson, Jose M. Rodriguez-Llanes

**Affiliations:** 1. Monash University Disaster Resilience Initiative, Melbourne, Victoria, Australia; 2. Western Australia Department of Health, Perth, Western Australia; 3. European Commission, Joint Research Centre, Ispra, Italy

**Keywords:** disaster, disaster data, disaster database, disaster measurement, risk reduction

## Abstract

**Objective:**

Disaster impact databases are important resources for informing research, policy, and decision making. Therefore, understanding the underpinning methodology of data collection used by the databases, how they differ, and quality indicators of the data recorded is essential in ensuring that their use as reference points is valid.

**Methods:**

The Australian Disaster Resilience Knowledge Hub (AIDRKH) is an open-source platform supported by government to inform disaster management practice. A comparative descriptive review of the Disaster Mapper (hosted at AIDRKH) and the international Emergency Events Database (EM-DAT) was undertaken to identify differences in how Australian disasters are captured and measured.

**Results:**

The results show substantial variation in identification and classification of disasters across hazard impacts and hazard types and a lack of data structure for the systematic reporting of contextual and impact variables.

**Conclusions:**

These differences may have implications for reporting, academic analysis, and thus knowledge management informing disaster prevention and response policy or plans. Consistency in reporting methods based on international classification standards is recommended to improve the validity and usefulness of this Australian database.

## Introduction

The frequency and severity of natural disasters is increasing, the effects of which are spread over greater geographical and increasingly populated areas. In the Australian context, the increasing risk to the built and natural environments related to increasing frequency and intensity of extreme weather events is described by the National Strategy for Disaster Resilience.^1^ Such risks have been realized by vast bushfires which swept across multiple states causing widespread destruction on the east coast of Australia over the summer of 2019/2020. To empower principles of “building back better,” resiliency, and supporting future disaster risk reduction (DRR) efforts and policy interventions in this context, an accurate understanding of hazards, threats, risks, and vulnerabilities is required. Measurement and understanding of the impacts caused by disaster informs policy makers and operational decision makers on investment strategies related to disaster. However, disaster risk analysis varies between institutions, partly due to differences in how disaster threats are defined and measured, and thus quantified. Previous studies in Australia measuring heatwave, a common Australian hazard, have demonstrated challenges in standardization of terminology and definitions, as well as data collection.^2^

The main weakness with disaster data is the lack of standardized methodologies and definitions for the inclusion of disasters^3^ and robust impact measurement methodologies.^4^ Accurate accounting for disaster impacts is a critical aspect of improving disaster risk management, DRR, and resilience building.^5^ Historical data are commonly used by analysts to track disaster trends and causal factors both over time and geographically. At subnational levels, disaster databases provide key information to signal hotspots of hazard or risk and design locally tailored actions plans or investigate regional trends. They can also be used to monitor progress in effectiveness of government strategies to reduce disaster impacts on population health and the economy.

Demand for clear, accurate, and consistent reporting of economic impact related to disaster in Australia is driven primarily from government and academia/research. Whilst a number of individual hazard-specific reports have been produced to date, the only comprehensive national impact assessment of economic loss related to disaster in Australia was conducted by The Bureau of Transport Economics (BITRE; Canberra, Australian Capital Territory) in 2001.^5^

Other disaster databases include data from Australia such as DesInventar,^6^ Swiss RE: Sigma,^7^ and Munich RE: NatCat.^8^ In the Oceania region, reporting systems include the Australia Disaster Assist^9^ and the Insurance Council of Australia Catastrophe Database,^10^ which reports on insurance losses from 1967 to the present. At the time of writing, a new database has been developed using data from the Australian Institute for Disaster Resilience (Melbourne, Victoria, Australia) Knowledge Hub (AIDRKH) and is currently available on request.^11^

This paper provides a comparative, descriptive review of disaster hazards in Australia as measured by a domestic disaster database, The Disaster Mapper at AIDRKH, and an international one, the Emergency Events Database (EM-DAT), to gain new insights on its compatibility with international standards of disaster data classification, reporting, and access.

## Methods

Studies comparing databases on disaster losses have broadly differed in their methodological approaches,^3^ ranging from narrative descriptions to mixed methods analyses^12^ and expert assessments or systematic reviews.^13^ Methodologies must be adapted to the purpose of the particular study and other constraints. This study used a combination of qualitative (eg, disaster definitions) and quantitative methods (eg, number of disaster events) to conduct the presented comparisons.

A comparative descriptive review of a national and an international disaster database was undertaken to examine differences in disaster definition and data entry thresholds, classification, impact (human, economic, and contextual), as well as accessibility and data structure. The choice of these variables was based on the review of past efforts to compare disaster databases.^3,12,13^ The databases used in the comparative review were the Australian Disaster Mapper based at the AIDRKH^14^ and the EM-DAT database of the Belgium-based Centre for Research on the Epidemiology of Disasters (CRED; Brussels, Belgium).^15^ These were purposively selected as the aim of the study was to provide a detailed account of whether the Disaster Mapper fulfills international standards for disaster databases according to a recognized and long-lasting initiative.

To conduct these comparisons, relevant information was scrutinized and extracted, including data, definitions, and classifications of the abovementioned variables from the corresponding sections of both websites.^14,15^ Relevant data on disasters and their impacts were downloaded to compare disaster frequencies for comparable categories of disasters, whenever possible.

The AIDRKH Disaster Mapper contains information on disasters affecting Australia and some international disasters that have impacted Australians since 1869. The Disaster Mapper was designed to support and inform policy, planning, decision making, and practice in disaster resilience and is managed by the Australian Institute for Disaster Resilience on behalf of the Australian Government. The Disaster Mapper includes natural, technological, and human-caused events that have a significant impact on Australia and its population. It is presented as an interactive visualization tool of disasters in Australia, supported by the annual Major Incident Reports involving Australian fire and emergency services. Disaster Mapper is likely the most comprehensive, publicly available national dataset, according to authors’ knowledge.

In 1988, CRED created the EM-DAT with initial support from the World Health Organization (WHO; Geneva, Switzerland) and the Belgian Government. The EM-DAT houses international disaster impact data from 1900 to the present day. The objective of the database is to serve and support national and international decision making for disaster preparedness, vulnerability assessment, and prioritize resource allocation for disaster response. The EM-DAT is a world-recognized and internationally-standardized source for disaster data and widely used by the United Nations (UN), international organizations, politicians, and academia.

## Results

### Disaster Definitions

The United Nations Office for Disaster Risk Reduction (UNDRR; Geneva, Switzerland) defines a disaster as “a serious disruption to the functioning of a community, which causes human, material, economic, and environmental losses beyond a community’s ability to cope.”^16^ The definitions of disaster used by each of the investigated databases are shown in Box 1. The definitions of disaster in the Disaster Mapper and EM-DAT are, with their lexical differences, well-aligned. They provide a clear understanding that disaster is a situation exceeding or overwhelming available resources at a certain level of aggregation, social or geographical, causing personal and/or material damage, and requiring more resources than those available at the affected communities.

Box 1. Disaster Definitions in EM-DAT and Disaster Mapper

**Table:** 

DISASTER MAPPER	EM-DAT
A disaster, as defined by the Australian Institute for Disaster Resilience, is a serious disruption to community life which threatens or causes death or injury in that community and/or damage to property which is beyond the day-to-day capacity of the prescribed statutory authorities and which requires special mobilization and organization of resources other than those normally available to those authorities.^14^	EM-DAT defines a disaster as: “A situation or event which overwhelms local capacity, necessitating a request to a national or international level for external assistance; an unforeseen and often sudden event that causes great damage, destruction, and human suffering.”^15^

### Disaster Database Entry Thresholds

Disaster databases apply criteria related to their respective definition of disaster that prescribe which events do and do not get recorded. Table 1 shows the entry criteria used by the two examined databases in this research. The mortality entry threshold, one consistently used across databases, was lower in the Disaster Mapper compared to EM-DAT, which could yield an increased ability to report disasters in the former.

**Table 1.** Disaster Database Entry Thresholds and Summarized Recorded Variables

	Disaster Mapper	EM-DAT
Disaster Entry Thresholds	A disaster, as defined by Disaster Mapper, is three or more deaths; or 20 injuries or illnesses; or significant damage to property, infrastructure, agriculture, or the environment; or disruption to essential services, commerce, or industry at an estimated total cost of A$10 million or more at the time the event occurred; and include event occurring in Australia or directly impacting on Australians.^10^	A disaster, as defined by the EM-DAT database, is ten or more people reported killed, or one hundred or more people reported affected, or declaration of a state of emergency or a call for international assistance.^11^
Reported Variables (Impact and Context)	Disaster Mapper database entries include date of event (Temporal Coverage 1753 – 2014), location (state and country), and disaster category. Narrative text accessible per event online contains additional information contextual to the event.^10^	The EM-DAT database includes information on the location (country or countries in which the disaster has occurred), the exact date of the disaster (start and end whenever possible), disaster categories, number of deaths, number of people injured, number of people homeless, number of people affected, and estimated economic damage.^11^

Abbreviation: EM-DAT, Emergency Events Database.

### Disaster Classifications

The Hazard and Peril Glossary is used for describing and categorizing disasters in the EM-DAT database, shown in Table 2.^17^ The Disaster Mapper database does not contain specific disaster definitions, yet it includes 17 disaster categories, which at times were found to be different compared to those in EM-DAT. Table 2 presents the disaster categorizations in both databases, in which EM-DAT contains more sub-categories of disasters. A notable difference is the use of a category labelled “environment” (Disaster Mapper) to classify extreme temperature and droughts (EM-DAT).

**Table 2.** Comparison of Disaster Classifications in EM-DAT and Disaster Mapper

CRED Emergency Events Database (EM-DAT)				Disaster Mapper
Disaster Group	Disaster Sub-Group	Definition	Disaster Main Type	Disaster Category
Natural	Geophysical	A hazard originating from solid earth. This term is used interchangeably with the term geological hazard.	Earthquake	Earthquake
			Mass Movement (dry)	Landslide
			Volcanic Activity	
	Meteorological	A hazard caused by short-lived, micro- to meso-scale extreme weather and atmospheric conditions that last from minutes to days.	Extreme Temperature	Environment
			Fog	
			Storm	Storm
				Cyclone
				Tornado
	Hydrological	A hazard caused by the occurrence, movement, and distribution of surface and subsurface freshwater and saltwater.	Flood	Flood
			Landslide	Landslide
			Wave Action	Tsunami
	Climatological	A hazard caused by long-lived, meso- to macro-scale atmospheric processes ranging from intra-seasonal to multi-decadal climate variability.	Drought	Environment
			Glacial Lake Outburst	
			Wildfire	Fire – Bushfire
	Biological	A hazard caused by the exposure to living organisms and their toxic substances (eg, venom, mold) or vector-borne diseases that they may carry. Examples are venomous wildlife and insects, poisonous plants, and mosquitoes carrying disease-causing agents such as parasites, bacteria, or viruses (eg, malaria).	Epidemic	Health
			Insect Infestation	Biosecurity
			Animal Accident	
	Extra-Terrestrial	A hazard caused by asteroids, meteoroids, and comets as they pass near-earth, enter the Earth’s atmosphere, and/or strike the Earth, and by changes in interplanetary conditions that effect the Earth’s magnetosphere, ionosphere, and thermosphere.	Impact	
			Space Weather	
Technological	Industrial Accident		Chemical Spill	Industrial
			Collapse	
			Explosion	
			Fire	
			Gas Leak	
			Poisoning	
			Radiation	
			Oil Spill	
			Other	
	Transport Accident		Air	Transport
			Road	
			Rail	
			Water	Maritime/Coastal
	Miscellaneous Accident		Collapse	Industrial Fire - Urban
			Explosion	
			Fire	
			Other	Criminal Other Disasters

Abbreviation: EM-DAT, Emergency Events Database.

### Impact Variables

The National Disaster Resilience Strategy endorses the consideration of risk and risk treatment across social, built, economic, and natural environments of a community.^1^ The Australian Institute for Disaster Resilience describes these four as recovery impact environments in the National Disaster Risk Reduction Framework.^18,19^ When planning for community needs, this framework guides planners on the interdependency of the four environments in considering and coordinating interventions.

Event impact variables provided by the Disaster Mapper vary between event type; however, deaths and injured due to an event are commonly reported in narrative text related to the event when accessed individually online. Event impact data of both databases are shown in Table 3.

**Table 3.** Disaster Impact Variables in EM-DAT and Disaster Mapper

CRED Emergency Events Database (EM-DAT) Impact Data Categories			Disaster Mapper Environments
Impact	Health	Death	Social Environment
		Missing	
		Total Deaths (deaths + missing)	
		Injured	
		Affected	
		Homeless	
	Economic	Total Estimated Damages (in 000US$ current value)	Economic Environment
		Reconstruction Cost (in 000US$ current value)	
		Insured Losses (in 000US$ current value)	
	Disaster	Sectors Affected by the Disaster (Animals, Industry, Electricity, Water Supply/Sanitation, Communications, Cultural Infrastructure, Transportation, Other)	Built Environment
		Infrastructure (infrastructure damaged or destroyed by the disaster, given in absolute values or percentages)	
		Comments (all other relevant information)	Natural Environment
		Other	

Abbreviations: CRED, Centre for Research on the Epidemiology of Disasters; EM-DAT, Emergency Events Database.

### Context Variables

Raw data in EM-DAT database contain further, context-related information not visible via the online portal. These context variables are described in Table 4.

**Table 4.** Context Variables in EM-DAT

Thematic Classifications	Variables
Geographical Information	Country (if a disaster has affected more than one country, there will be one entry for each country)
	ISO Code (International Organization for Standardization 3-letter code for each country)
	Region (as per the UN regional division)
	Continent
	River Basin (if flood event)
	Latitude/Longitude/Location (eg, name of a city, village, department, province, state, or district)
Temporal Information	Start Day/Month/Year
	End Day/Month/Year
	Local Time
Physical Characteristics	Origin
	Associated Disasters 1 and 2 (ie, landslide post-earthquake)
	Disaster Magnitude Scale and Value
	Other
Status	Aid Contribution: Total Amount (given in 000US$)
	OFDA Response
	Appeal for International Assistance and Date
	Declaration of Disaster and Date

Abbreviations: EM-DAT, Emergency Events Database; UN, United Nations; OFDA, Office of US Foreign Disaster Assistance.

All events recorded in the Disaster Mapper can be individually viewed and contain a brief narrative of the event and its impact from where some contextual variables could be potentially obtained. Annual major incident reports have been produced by AIDKH for the last three years based on database inputs.

### Database Accessibility

The EM-DAT database is accessed through an online portal requiring a username and password applied for through the CRED website. The EM-DAT database provides several standardized reports that can be generated and customized to region, country, and disaster. Advanced search functions on EM-DAT allow for specific event searches and automatic report generation, the data of which can be extracted. Access requires registration as a user.

Events recorded in the Disaster Mapper are publicly accessible and can be viewed as multiple or single disaster categories; however, event data can only be accessed per event and report or data extraction is not available through direct online access.

### Disaster Database Recorded Events

All disaster data were manually extracted from the Disaster Mapper database. As of November 1, 2019, a total of 416 events had been entered: 396 of these were events that occurred in Australia and 20 events that affected Australian nationals abroad occurred internationally. Table 5 shows Disaster Mapper events by category type in order of number of events (top 10) built using all available data from 1869 to 2019. Table 6 is a direct extract of data from EM-DAT, which shows Australian disasters from 1900 to 2019 also in order of number of events (top 10).

**Table 5.** Australian Disaster Mapper Disasters (Top 10 by Number of Events)

Australian Disaster Mapper Categories	Number of Events 1869-2019
Flood	80
Fire – Bushfire	65
Industrial	53
Cyclone	45
Storm	39
Health	34
Fire – Urban	20
Transport	19
Environment	14
Criminal	7

**Table 6.** EM-DAT Australian Disaster Events (Top 10 by Number of Events)

EM-DAT Disaster Type	Number of Events 1900-2019
Storm	107
Flood	64
Wildfire	41
Transport Accident	23
Drought	11
Miscellaneous Accident	8
Extreme Temperature	7
Earthquake	4
Epidemic	2
Industrial Accident	2
Insect Infestation	2
Landslide	2

Abbreviation: EM-DAT, Emergency Events Database.

Whilst the original start date of recording of data differs between the two databases (1869 versus1900), there were only seven (7) events recorded in the difference between these time periods: five (5) floods and two (2) industrial accidents.^16^ Inclusion of these events does not substantially alter the proportionate difference in numbers of events or make the databases more comparable. The observed trends in these comparisons were overall as expected. With increased sensitivity in Disaster Mapper, this database recorded increased number of floods and wildfires, with very substantial differences for epidemic outbreaks and industrial accidents. Storms and droughts presented more comparable numbers across databases, while other categories could not be assessed from Table 5 and Table 6.

Furthermore, the Disaster Mapper contains an “Other” category where two recorded events have been entered. Box 2 shows a summary of these events. In contrast, the EM-DAT database does not record war or conflict-related events.

Box 2. Australian Disaster Mapper Database “Other” Events

**Table:** 

August 5, 1944	A Prisoner of War (POW) attempted mass escape in Cowra, New South Wales resulted in 235 deceased and 108 injured
February 19, 1942	Wartime bombing in Darwin, Northern Territory resulted in 243 deceased and 400 injured

The EM-DAT database does include a category of miscellaneous accident in the technological category, which also holds an “other” selection. Only one event in this category has been captured in the EM-DAT database relating to an event occurring in 1990 that resulted in 25 deaths. Following inquiry with the database management team, no detailed information was available to describe this event.

Other notable differences include the categories of “health” and “criminal” in the Knowledge Hub. Further investigation of the “health” category revealed details of events such as heatwaves, food poisoning, listeria, gastroenteritis, coral poisoning, poliomyelitis, bubonic plague, Spanish flu, and bird flu (H1N1) events. These events are captured and recorded in different categories listed in EM-DAT (ie, biological, meteorological).

Investigation of the “criminal” category in the Knowledge Hub found ten (10) events, five (5) of which were terrorist events that occurred overseas. The EM-DAT database does not include terrorist attacks or other criminal-related events as a disaster category.

The Australian Institute for Disaster Resilience has published three (3) reports based on events recorded in the Knowledge Hub. Titled “Major Incidents of the Year,” reports for 2016-2017, 2017-2018, and 2018-2019 have been produced. Each report provides an overview of major incidents that have involved the fire and emergency services sector during the corresponding financial year. The intent of the reports is to examine incidents identified by the sectors that were of significant impact or consequence for fire and emergency services. The reports are not a review of all incidents occurring during the period defined and are intended to provide key insights related to the events described. These publications provide a user-friendly resource for emergency service operators to engage with lessons learned in their field.

Currently, CRED provides a biannual newsletter based on EM-DAT data, an Annual Disaster Statistical Report, and CRED Crunch, a newsletter published typically every three to six months. The newsletter focus is broad and reflective of international disasters. On occasion, EM-DAT data are used for international reports with a thematic focus.

## Discussion

This study compared essential characteristics of the EM-DAT database and the Disaster Mapper disaster impact databases focusing on records from Australia. Whereas both databases emerge from similar definitions of disasters, substantial differences were found. A lack of some categories and general absence of definitions were noted when comparing them. An even more important aspect was the lack of a clearer data structure to report contextual and impact variables. Disaster Mapper considered war-related events not considered in EM-DAT, and considered the environmental impact of disasters and not just the direct human impacts.

Entry criteria for an event in the Disaster Mapper appears to align with the published criteria for the Australian Disasters Collection by including “natural, technological, and human-caused events that have a significant impact on Australia and its people.” It is not clear how significance is calculated for international events, as other impacts on Australian Nationals abroad, such as the downing of Malaysia Airlines Flight 17 (MH17) on July 17, 2014 resulting in the death of 283 passengers, including 38 Australians, has not been included. Additionally, other historic natural disaster impacts, such as the volcanic eruption in Papua New Guinea (then a territory of Australia) that resulted in 4,000 deaths, are missing from the Disaster Mapper.^20^ It is unclear how or why events were selected for inclusion and others were not.

The EM-DAT database does not record war or conflict-related events. Alternately, CRED has identified events related to the impact of war or conflict as “complex emergencies” from which the Complex Emergencies Events Database (CE-DAT) was developed and captures humanitarian emergency impact data. The intent of CE-DAT was monitoring and evaluation of the health status of populations affected by complex emergencies. The CE-DAT was initiated in 2003 to predominantly measure mortality and malnutrition from surveys conducted in humanitarian crises. The CE-DAT database is not currently operational.

The EM-DAT possesses a hierarchical clustering of main disaster categories and sub-categories, which could be used by Disaster Mapper to improve its classification structure and assist in addressing absence of categories for mass movement, meteorites, and volcanic activity.

Further comparisons of the datasets are challenging due to accessibility options. Whilst EM-DAT enables spreadsheet downloading of the data, Disaster Mapper data require manual extraction and configuration into usable tables. Overall, the differences in data collection and functionality between the two databases limit meaningful comparison. For data users, this can potentially challenge database utility for policy guidance, development, and decision making.

Consistent with the study results here, internationally led research has compared disaster loss databases in efforts to improve understanding of disaster impact. A report commissioned by the United Nations Development Program (UNDP; New York USA) reviewed country and regional disaster databases and highlighted that in the Asia Pacific region, of 19 different national databases that were identified, five (including Australia) had stand-alone methodologies for disaster event capture and recording.^21^ The remainder used DesInventar definitions and classifications. Disaster information captured by DesInventar format databases include: type of event, province/State, district, date, location, deaths, missing, injured, affected, victims, evacuated, relocated, houses damaged, houses destroyed, crops and woods (hectares), livestock (lost), educational centers, hospitals, loss value in local currency and USD (calculated according to the exchange rate on the date of the disaster), roads affected, and other data fields up to a maximum of 17 additional parameters (including data sources for each of the records).^6^ The Australian database examined by UNDP was reported as including event title, zone, region, category, start date, end date, dead, injured, and the insured total losses due to the disaster itself. Interestingly, the reference used by the report related to Australian disaster data is the Emergency Management Australia Disasters Database.^22^ The data set was created on May 2, 2014 and last updated on December 16, 2016 as a CSV format list of all Australian Emergency Management Knowledge Hub disaster events, including disaster category, impacts, and geographic coordinates. The dataset is publicly available for download but shows a difference in disaster events recorded (a total of 674) compared to Disaster Mapper.

Key findings noted by the UNDP report included opportunities for improvement in currency (up to date information), completeness (data gaps), quality assurance (having a documented quality control procedure), applications (use of the dataset for research or policy support), accessibility (having open access), and standardization (using consistent methodology). The report endorsed recommendations to improve disaster loss databases in respect to these criteria and defined the ideal loss and damage database as “one that is sustainable, continuous, credible, publicly accessible, quality assured, and applied for decision making.”^21^ These recommendations are consistent with findings of an investigation into disaster data interoperability in Europe by Migliorini, et al who noted a lack of long-term DRR activities related to data capture and usage.^23^

The EM-DAT is one of very few global disaster event databases. The EM-DAT, along with other international databases, relies predominantly on media sources, international organizations (ie, UN, Red Cross), and/or non-governmental organization reports, resulting in a lack of readily available access to event data that national services possess. Consistent standards of data capture and shared access may enhance research capability to investigate disaster impact events. Findings from De Groeve, et al recommended guidelines and standards for data collection and recording, with a focus on human and economic losses, to enable data sharing in a comparable way.^13^

An investigation into decision making related to disaster resilience in Australia conducted by Deloitte found that gaps existed across categories of data and that “significant barriers exist to the better provision, sharing, and quality of natural disaster data sets.” Recommendations noted by Deloitte include a more coordinated approach to natural disaster data to reduce cost and support the quality of research activities and decision making related to resilience investments, and reduce the duplication of data collection and analysis.^24^

In a study using both CRED data and Knowledge Hub entry data, Bradt, et al sought to determine the profile of Australian Disaster since 1900. Large variations in data capture and classification were also identified by the author. To account for this and enable a sharper analysis, a methodology was developed by the author and applied using additional criteria in order to exclude events not deemed of national significance.^25^

The collection of accurate disaster loss information is of relevance to many stakeholders. Hallegatte, et al reported national and subnational levels of government, the insurance sector, the private sector, and the local and international community as having invested interest in disaster loss information to guide risk plans and actions.^26^

As described by De Groeve, et al, one of the main sources of incompatibilities between databases is the lack of precise and agreed definitions of hazards and loss indicators.^13^ The analysis here is coincident with the above statement. Enhancement of the Australian database could be achieved through adoption of the Integrated Research on Disaster Risk Programme hazard and peril classification, which is widely adopted across national and international databases. This classification distinguishes three levels: the event family (the most generic), the main event type, and peril (the most specific).^17^ These findings are consistent with the outcomes of a review of selected disaster databases by Tschoegl, et al who conducted a high-level overview of international and national disaster database methodologies.^27^

Australia is a signatory to the Sendai Framework for Disaster Risk Reduction, Priority 2, of which is “Strengthening disaster risk governance to manage disaster risk.”^28^ Differences in hazard definitions, lack of certain hazard categories, and varying entry criteria may result in inclusion of events in one dataset that may not be included in the other. This, in turn, can alter perception of, and decision making related to, risk and vulnerability to hazards or biased disaster response. Addressing this issue is of particular relevance as Australian disaster reporting seeks to move from a response to a prevention approach.^18^

There is no national strategy, organization, or capability to systematically capture, measure, and evaluate disaster event occurrence, impact, and outcomes and from this analyze and implement lessons and findings into policy or practice. The recently released report of the Royal Commission into National Natural Disaster Arrangements has recommended improvements in national practices of disaster data collection. In particular, implementation of harmonized data governance and national data standards and development of consistent data standards to measure disaster impact.^29^

The findings of this paper identify opportunities for improvement. This includes a recommendation of review of Australian disaster database hazard classification and definitions in alignment with the Integrated Research on Disaster Risk Programme hazard and peril classification. Further to this, standardization and systematic reporting of disaster data utilizing an agreed, fixed data structure including context and impact variables internationally is recommended. Finally, to enhance utility for generation of rapid situation reports or customized reports online, disaster database data extraction capability is recommended.

## Limitations

This study is not exempt from limitations, including the comparison of only two databases. Limited accessibility was observed at the time of the study. It should be noted that EM-DAT has enabled public access after this analysis was completed. This research was conducted from an Australian perspective and may lack validity outside of that perspective.

## Conclusion

This paper provides a comparative analysis of disaster hazard and threat data of Australian events as measured by The Australian Disaster Mapper and the CRED EM-DAT database. Differences in categorization and classification were identified, which may have implications for reporting and analysis. Further investigation to understand how significant events are identified for inclusion in disaster categories, and how their inclusion impact decision making for DRR activities in Australia, is warranted. Consistency in reporting methods based on international classification standards is recommended to improve the validity and usefulness of this Australian database.
